# Workplace violence in the ambulance service from the offender’s perspective: a qualitative study using trial transcripts

**DOI:** 10.1186/s12873-025-01232-w

**Published:** 2025-05-13

**Authors:** Magnus Viking, Karin Hugelius, Erik Höglund, Lisa Kurland

**Affiliations:** 1 Department of Ambulance Care, Faculty of Medicine and Health, Örebro University, Örebro, Sweden; 2 Faculty of Medicine and Health, Örebro University, Örebro, Sweden

**Keywords:** Aggression, Ambulance, Ambulance nurse, Ambulance service, Offender, Trial transcripts, Qualitative, Workplace violence.

## Abstract

**Background:**

Workplace violence is a widely recognised problem within the ambulance service context. The causes of workplace violence have often been attributed to patient- or situation-related risk factors. However, there is a lack of research on workplace violence from the offender’s perspective.

**Aim:**

To explore workplace violence directed toward ambulance services from the offender’s perspective.

**Methods:**

An explorative qualitative study was conducted using inductive thematic analysis of trial transcripts from cases tried in court between 2013 and 2023. Plaintiffs in these cases were ambulance personnel or the ambulance service itself. Offenders were those convicted of committing or attempting any of the following acts: threats, theft, assault, molestation or murder.

**Results:**

Twenty-three trial transcripts were analysed, and four themes were found: (I) *the offender was misunderstood*, which included communication problems and other misunderstandings; (II) *the offender was disrespected*, which described perceived unprofessional behaviour and unpleasant or painful treatment by ambulance personnel; (III) *the offender was vulnerable*, which described the state of the offender (i.e., being under the influence of alcohol or drugs); and (IV) *the offender had unmet expectations*, which included perceived unreasonable waiting time and conflicting expectations of ambulance care.

**Conclusion:**

The analysis of trial transcripts revealed four themes from the offender perspective: feelings of being misunderstood, disrespected, vulnerable, and having unmet expectations. It is important to view these results critically, as they are based on trial transcripts in which the offender was found guilty of a crime and may have been attempting to defend his or her actions during the trial. Despite this caveat, healthcare professionals need also to recognise that their behaviour may influence the risk of workplace violence. This knowledge can be harnessed to develop training programs for ambulance personnel.

## Background

Workplace violence is a worldwide problem within the ambulance service and affects both service delivery in addition to leading to, consequences for the ambulance personnel [1–6]. Workplace violence is defined as any harassment, intimidation, threat of violence, act of violence, or threatening behaviour that occurs toward healthcare personnel with the intent to cause harm [7].

The incidence of workplace violence directed towards ambulance services or personnel varies, ranging from 0.4 to 5% of all ambulance missions [8–13]. The most common type of workplace violence against ambulance personnel is non-physical violence (i.e., verbal threats or intimidation), followed by physical violence, such as assault [8–10, 14]. In addition to the risk of physical injury [4, 8], ambulance personnel have been shown to experience psychological distress and burnout as a consequence of workplace violence [15, 16]. In addition to the direct risk it poses to ambulance personnel, workplace violence can negatively impact the quality and delivery of care [17].

Previous research on workplace violence within the ambulance service has shown that most frequently, the patient is the offender [1, 8, 10], is most often a man between the ages of 18–39, and is commonly under the influence of alcohol or drugs and suffering from mental health issues [8, 10, 18]. The ambulance personnels’ physical working environment makes them vulnerable to workplace violence, e.g. when caring for patients in the limited space of the ambulance [1], or because they carry prescription drugs which could be of interest to some offenders [1]. Research on workplace violence toward ambulance personnel is sparse and predominantly describes factors where the patient is the offender. However, little is understood to why offenders expose ambulance personnel to workplace violence.

Moreover, despite knowledge of these risk factors, prior studies do not explain why not all patients with these risk factors do not expose ambulance personnel to workplace violence. The perspective of the offender has been little studied and is needed to better understand the complexity of workplace violence directed towards ambulance personnel, and its potential triggers. Therefore, the aim of the current study was to explore workplace violence directed toward ambulance services from the offender’s perspective.

## Methods

### Design

An inductive explorative qualitative study was conducted using the analysis of court trial transcripts.

### Data collection procedures

Trail transcripts were used to analyse workplace violence from the offenders´ perspective. In Sweden, a trial transcript is a document created by the court clerk during a trial and includes the statements of the involved parties. Inclusion criteria for the trial transcripts were as follows: (1) transcripts from Swedish district courts. (2) from a trial that resulted in an individual being convicted of making threats, theft, assault, molestation, murder, or attempts at any of these offenses (3) trial transcripts from cases in which ambulance personnel or the ambulance service served as the plaintiff. The plaintiffs in the trial transcripts were either on-duty ambulance personnel or the ambulance service itself.

An offender was defined as a person who committed and was convicted of an offense accordingly to the above mentioned crimes [20]. In this case, they are also referred to as convicted offenders.

Trial transcripts from 2013 to 2023 were included.

Data collection was conducted in two steps:

(I) In the first step, requests were made to procure all trial transcripts from 2013 to 2023 regarding “crime against a person,” according to Chaps. 3 and 4 of The Penal Code (‘Brottsbalken’), or “sabotage against emergency service activities” [21]. Trial transcripts were requested from a total of 11 district courts (Attunda, Nacka, Solna, Stockholm, Södertälje, Södertörn, Malmö, Göteborg, Uppsala, Västmanland, and Örebro). Since the district courts were not able to apply more specific search terms, this method ensured broad inclusion of data retrieval. A trial transcript was included only if the plaintiff was a member of ambulance personnel or the ambulance service itself. Limitations in some of the court data systems prevented them from discerning which transcripts to send, and others wanted financial compensation for retrieving the requested data. As such, only seven district courts (Solna, Södertörn, Södertälje, Stockholm, Malmö, Nacka, and Örebro) were able to provide the requested transcripts. Trial transcripts containing the word “ambulance” were read in their entirety by the first author (MV). If the trial plaintiffs were ambulance personnel or the ambulance service itself, the transcript was considered to have met the inclusion criteria.
  This data collection resulted in 15,107 trial transcipts collected on requests from district courts in step 1. Of these, 14,057 were excluded for not including the word “ambulance”, leaving 1,037 trial transcripts. 13 of the remaining trial transcriptsy included ambulance personnel or ambulance service as plaintiffs and were included in the study.

(II) In the second step, the first author (MV) conducted a manual internet search to find news articles on workplace violence against ambulance personnel. The internet search was performed using Google and Swedish terms for “ambulance”, “workplace violence”, “attacked”, “ambulance personnel” and “verdicts” in different combinations. The search was conducted between December 1st 2024 and February 1st 2025. Trial transcripts were subsequently requested from the corresponding district courts based on the date and location of the incident or the names of the involved parties presented in the articles. The manual search contributed by identifying cases of workplace violence directed towards ambulance personnel that were deemed to be missed due to technical and economic restraints when requesting sets of trial transcripts from several district courts. These issues were no longer a constraint when only one specific trial transcript was requested.

**Fig. 1:**
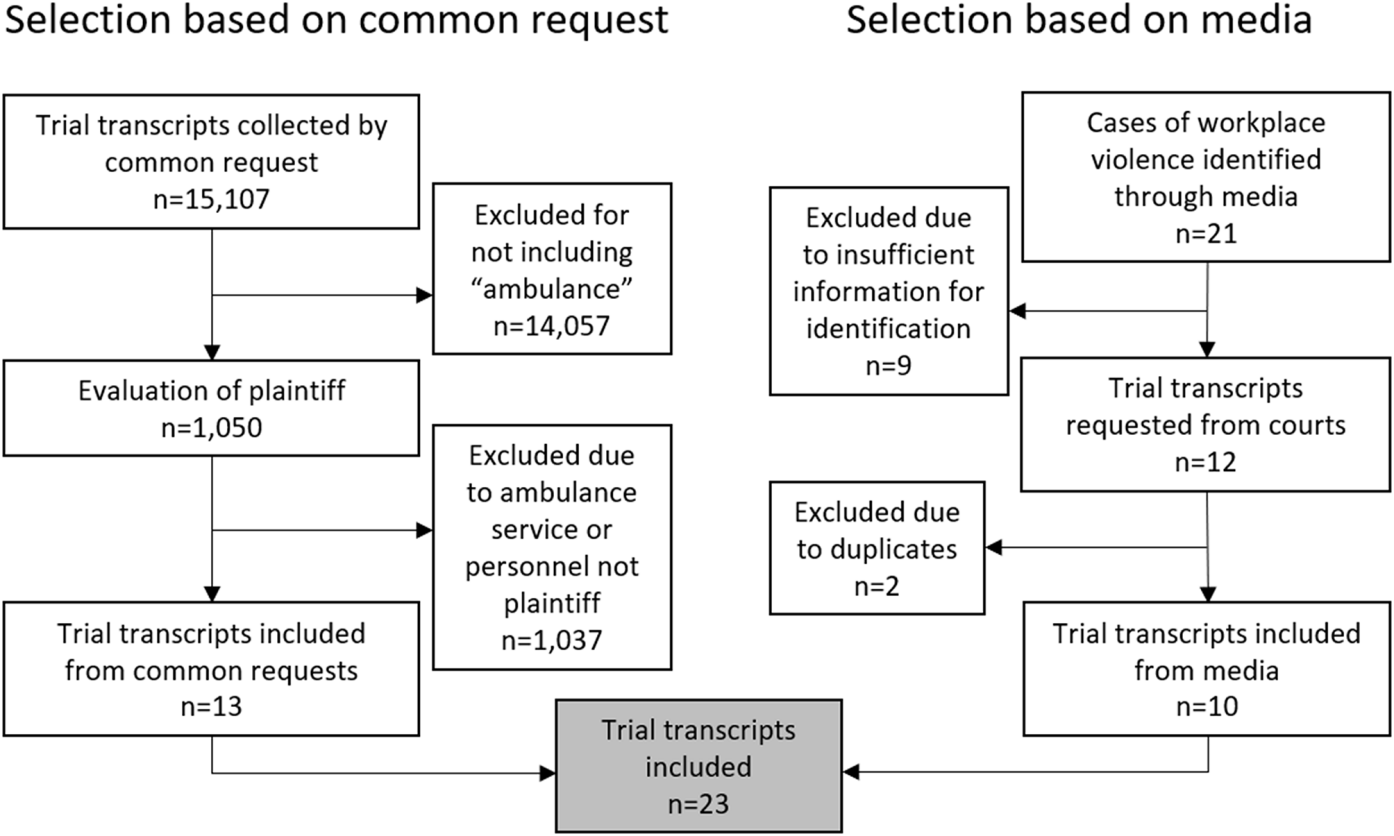
Flowchart of included and excluded trial transcripts

The step II rendered 21 cases of workplace violence that were identified through media. 9 of these were excluded due to a lack of information for identification, and 2 were excluded due to duplicates with trial transcripts included from step 1. Finally, 10 trial transcripts were included in the study adding up to the total of 23 trial transcripts included from step 1 and step 2 (see Fig. 1). Table 1 displays the offenders’ background information. Of the offenders from the 23 trials, 18 were men and 6 were women; one trial transcript included two offenders.

**Table 1:**
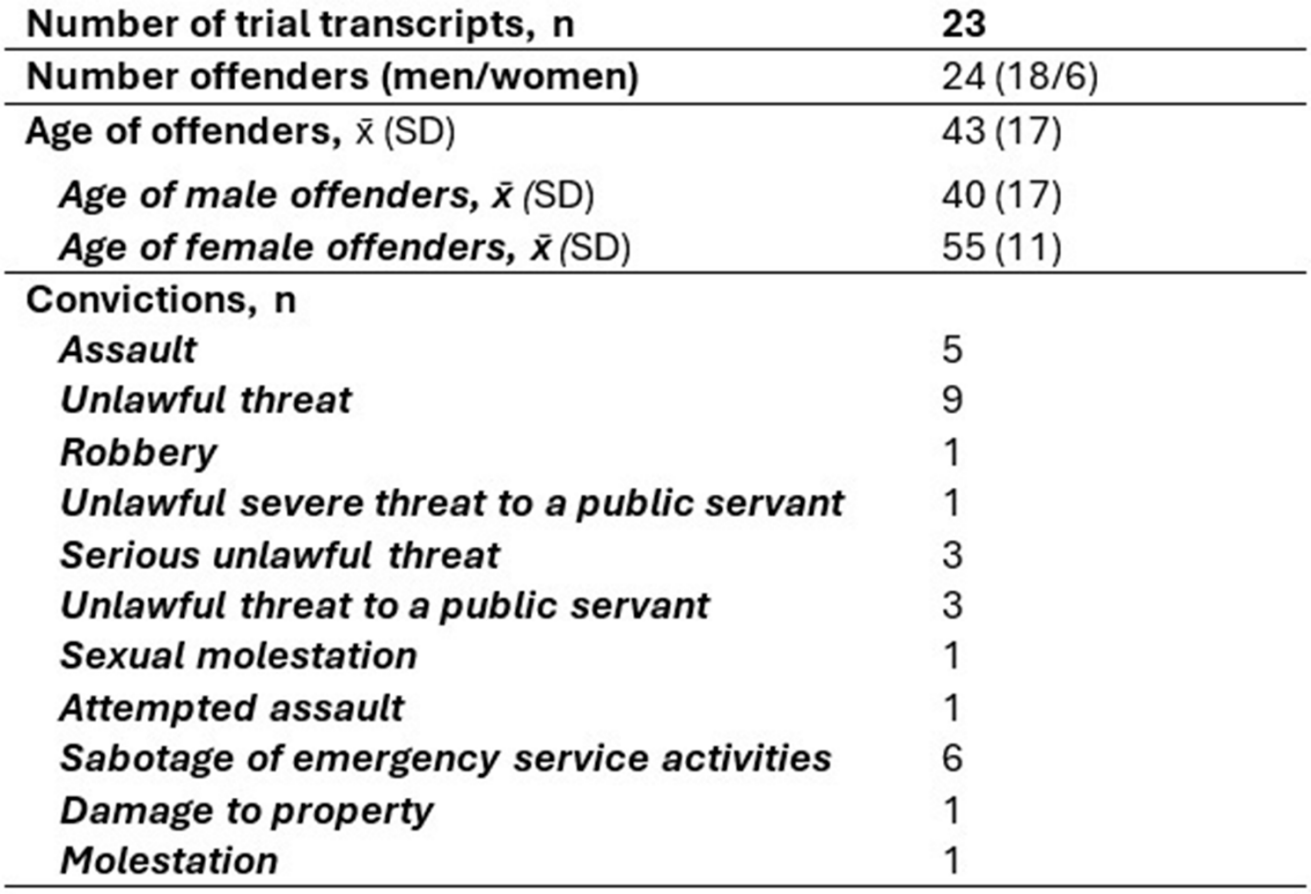
Description of the offenders and types of crimes

### Analysis

Inductive thematic analysis [22] was performed using the selected trial transcripts. Focus was placed on the offender’s testimony of the sequence of events, as presented in the trial transcripts. All trial transcripts—regardless of what crime the offenders were convicted of—were analysed in their entirety. The data-driven analysis was conducted in six steps [22]. First, to gain familiarity with the content, the first author (MV) carefully reviewed the entire text of each trial transcript multiple times. Next, codes were generated by sorting similar extracts from the text, collected in an Excel file (Microsoft^®^ Excel^®^) (MV). Third, patterns and relationships among the codes were identified and then grouped into overarching themes. All the authors participated in this step. A visual representation was created to illustrate the relationships between the codes and themes. Fourth, the authors discussed and assessed the codes, themes, and their relationships to each other against the original data. In the fifth step, the themes were defined. Finally, the manuscript was written [22]. The first author led the analysis process with support from the coauthors (KH, EH, and LK). The authors collectively discussed the final interpretation of the results in relation to the original data.

To illustrate the results, direct quotations of the offenders’ statements (in italics and enclosed in quotation marks) or summaries of statements present in the trial transcripts (in italics without quotation marks) are presented in the results section.

## Results

**Fig. 2:**
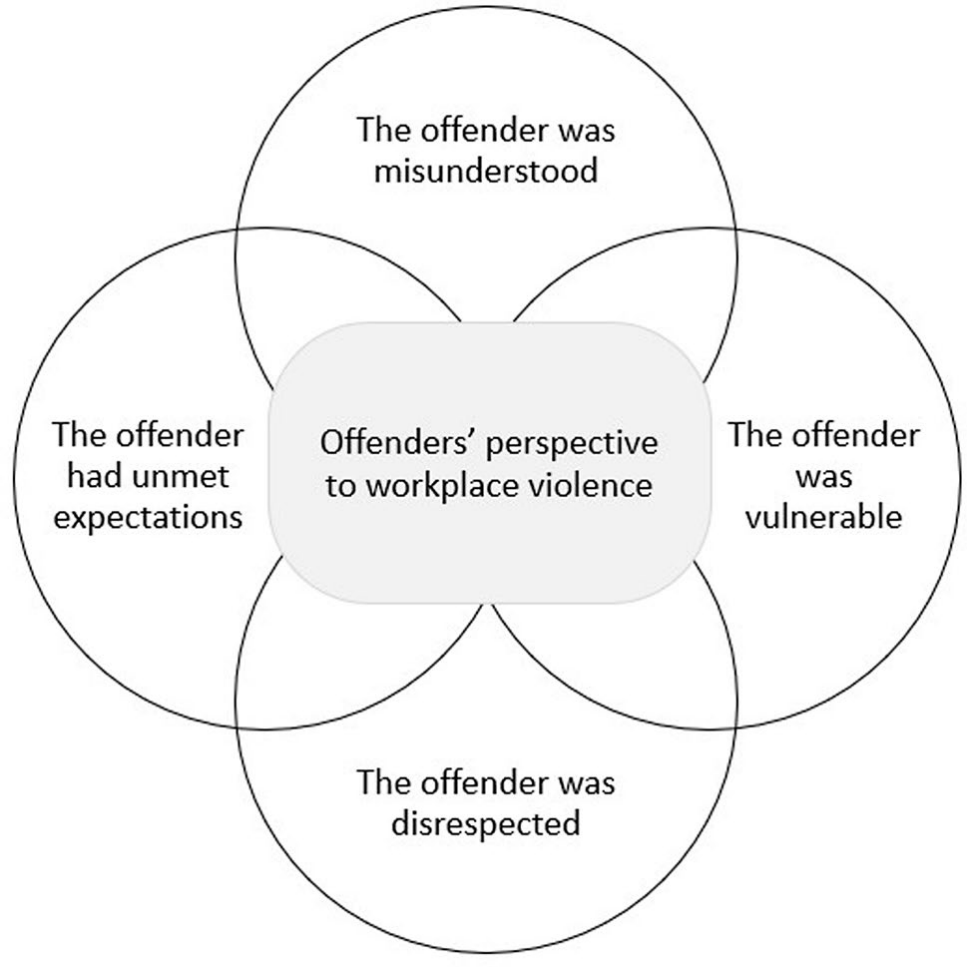
Themes reflecting workplace violence against ambulance service from the offender. Themes are presented in the circles in an interactive role

The analysis revealed four themes among the offenders’ explanations for their workplace violence incidents against the ambulance service: *the offender was misunderstood*,* the offender was disrespected*,* the offender was vulnerable*, and *the offender had unmet expectations.* Figure 2 presents an overview of the themes and their relationships.

### The offender was misunderstood

The theme of the offender being misunderstood includes issues related to *communication difficulties*, *not knowing the consequences*, and *not knowing what was happening*. Language differences between the offender and ambulance personnel led to misunderstandings and emotional distress on the offender’s behalf.

*Inside the ambulance*,* he felt respiratory distress because he had such severe bleeding. In Persian*,* he told the ambulance personnel several times to stop the car*,* but the ambulance personnel didn’t understand and only shook his head.*

- Trial transcript 3.

Other examples of communication difficulties occurred when the offender was under the influence of alcohol or drugs, which impaired their ability to express themselves.

*The offender explained that he was upset since he had previously been beaten by a security guard and thought that he was [once again] being abused by a security guard.*

- Trial transcript 10.

Misunderstanding also occurred when the offender mistook the ambulance personnel member for someone else.

### The offender was disrespected

Being disrespected included the issue of being subjected to *unpleasant treatment* or *discourtesy*. Examples of unpleasant treatment included ambulance personnel yelling, banging on the door, abruptly removing a blanket from the offender’s bed without warning, or abrupt or rough attempts to assess the offender’s level of consciousness. These situations occurred predominantly when the ambulance personnel were the first responders on the scene.

*The ambulance personnel knocked on the door but got no answer. They opened the door and entered the residence. They found the patient on the bed under a blanket and when they woke the patient up*,* she got irritated and kicked in the direction of the ambulance personnel.*

- Trial transcript 11.

Additionally, the offender could perceive physical examinations as painful or uncomfortable. This perception could be due to underlying medical conditions that cause pain or even the requirement to undress for a physical examination.

The offender explains that he hit the ambulance personnel without thinking when they grabbed his left arm, where he has a prosthesis. He has told the court that he has phantom pains in his left arm, which makes him twitch when someone grabs the left side of his body and his left arm.

- Trial transcript 18.

An offender might also be triggered into workplace violence when ambulance personnel threaten to involve the police. An example of this was reflected in a case in which a patient with a non-conclusive condition was threatened to be forcibly transported to the hospital. Another example reflected the unprofessional behaviour of an ambulance personnel member entering the offender’s private residence without permission. This can also be seen in cases in which the offender was unaware that the ambulance was even called.

The offender has stated that the reason he uttered the threats was because he felt sad and angry, and that he just wanted to get out of there and that he can’t cope in situations where he feels crowded.

- Trial transcript 4.

The presence of many people, such as ambulance personnel and police officers, contributed to the offender feeling overwhelmed and in a vulnerable position. This, in turn, triggered the offender’s aggressive and violent behaviour.

### The offender was vulnerable

The theme of being vulnerable included issues related to the *use of alcohol or drugs*,* chronic pain*,* mental health issues* or *stress*. Many offenders expressed that their disorientation was caused by mental illness—such as psychosis and suicidal tendencies—or the influence of alcohol or drugs. From these offenders’ perspectives, the conditions explained their aggressive behaviour toward ambulance personnel.

The offender stated that he had been drinking alcohol and that he has little memory of what took place in the residence. He stated that he suspects that he had [an episode of] psychosis, that he remembers a tug of war over the rifle but he does not know with whom, and that he has no memory of any ambulance personnel being there.

- Trial transcript 2.

Other circumstances that offenders expressed as a trigger for their violent behaviour were long-term pain, a poor financial situation, or being under considerable stress due to severely ill relatives or other external causes.

*At the time of the event*,* the offender explained that he was in a bad situation. There were threats against him and his family; he had an escalating heroin addiction and needed both drugs and money. He came up with the idea of robbing an ambulance personnel member of their medical bag.*

- Trial transcript 21.

In some situations, the offender had the intention of committing suicide, either through self-inflicted means or by deliberately instigating a situation that could lead to his or her death. As a result, they deliberately used weapons or other acts of physical violence against ambulance personnel to provoke a reaction.

*The offender opened the door for the ambulance personnel with a knife in her hand*,* threatening to kill anyone who attempted to come close to her. When the police arrived*,* they pulled their weapons. The offender kept being aggressive*,* […] repeating her threats and stabbing the door frame with the knife.*

- Trial transcript 20.

In one case, the offender was desperate for drugs or money. To gain access to drugs, the offender intentionally planned to threaten and rob ambulance personnel.

### The offender had unmet expectations

The theme of unmet expectations included issues related to having *different expectations* and prolonged *waiting times.* Ambulance personnel and patients—or their family members—may possess conflicting expectations of the nature and delivery of care, and this is another reason why workplace violence incidents can occur. In one situation, a patient’s relatives were prevented from ensuring adequate care and supporting them with language translation. Due to visitation restrictions, the relatives were not permitted to accompany their family member during the ambulance ride. This, in turn, provoked aggressive behaviour.

*The patient’s relatives demanded that one of them accompany the patient to the hospital*,* but the request was denied due to COVID-19 restrictions*,* [and this] led to aggressiveness towards the ambulance personnel.*

- Trial transcript 1.

Other triggers of aggressive behaviour included disagreements regarding the urgency of a medical condition or the need for emergency care. In addition, aggressive behaviour could be triggered in situations where the offender either felt forced to go to the hospital against their will or, contrary to their expectations, was denied transport to the hospital altogether.

*The offender’s father had been waiting for an ambulance for two hours and when his father stopped breathing*,* he drove him himself […]. [He] met an ambulance on the way*,* […] stopped it*,* and requested help. He was agitated and yelled that he would kill everyone in the ambulance*,* at the emergency call centre*,* and […] at the hospital.*

- Trial transcript 14.

Perceived long waits could also agitate the offender, causing frustration or disappointment. In one situation, the patient succumbed to their condition while waiting for the ambulance’s arrival. This caused great frustration to the patient’s relative, who the directed this violence and aggression toward the ambulance personnel.

### Comprehensive understanding

There are various reasons why aggressive behaviour can lead to violence toward ambulance personnel. From the offender perspective, it has been explained as arising from: misunderstandings between the offender and the ambulance personnel, the offender feeling disrespected by the ambulance personnel, the offender being in a vulnerable state, or a mismatch in expectations regarding the care provided. In some cases, several underlying conditions or factors could coexist. For example, intoxication from alcohol or drugs or severe mental illness can hinder both communication and understanding of the ambulance personnel’s intentions. This, in turn, can lead to misunderstandings and feelings of being disrespected. Sometimes, the offender may be unable to clearly explain these situations due to factors such as blackouts or intoxication. They may perceive these situations as unintentional, impulsive events. However, not all instances of workplace violence occur unintentionally. Some incidents are planned, for example, with the aim of acquiring drugs or valuables. The offender may be experiencing stressful financial conditions, perhaps caused by an escalating drug addiction. This places the offender in a vulnerable situation, and they may turn to violence to cope.

## Discussion

The trial transcript analysis of offender experiences and explanations for workplace violence (measured as convicted crimes) revealed four themes: feeling misunderstood, feeling disrespected, feeling vulnerable, and having unmet expectations.

The offenders stated that they were misunderstood in the given situation. These patients were under the influence of alcohol or drugs at the time of the incident, posing additional communication challenges. It is well known that alcohol and drug use are contributing factors to workplace violence and that alcohol consumption increases aggressive behaviour [13, 23, 23]. The current study revealed that when the offenders were under the influence of alcohol or drugs, the risk of miscommunication between the patient and the ambulance personnel increased. Alcohol and drugs can decrease the ability to interpret voice and facial expressions [24], making it harder for a patient to understand ambulance personnel. Therefore, ambulance personnel should enhance their communication strategies with patients under the influence of alcohol or drugs. This can decrease misunderstandings and potentially mitigate the risk of workplace violence.

This study found that some offenders had been subjected to uncomfortable medical treatments and evaluations. This may have played a role in their perceptions; some offenders felt that the ambulance personnel had disrespected them. Offenders with long-term pain perceived specific treatments as uncomfortable. It is known that patients suffering from chronic pain believe that physical examinations increase their pain [25]. In this way, ambulance personnel should adopt a gentle and respectful approach and communicate with patients during patient assessments or treatments. This can lower the level of patient discomfort and potentially reduce the incidence of workplace violence against ambulance personnel.

The offenders perceived themselves as vulnerable when experiencing pain, stress, mental health issues, or the effects of alcohol or drugs. These results corroborate existing research, which reports that the risk of workplace violence toward healthcare personnel increases when a patient has specific personality traits or impaired social cognition, is under the influence of alcohol or drugs, is in pain, has mental issues, or is in a precarious situation [26]. It should be noted that this study does not evaluate the offenders’ personalities or social cognition abilities. Thus, a direct comparison of these factors cannot be made. In the context of ambulance services, such investigations could yield further insights into the vulnerabilities of patients and their connection to workplace violence against ambulance personnel.

Previous studies on workplace violence in emergency departments highlight the significance of unmet expectations (e.g., from patients, their family members, and healthcare personnel). Similar to the results of this study, differing views on expected and provided care have been seen to trigger workplace violence [27]. To reduce mismatches in expectations and facilitate a sense of patient safety, it is important that patients receive sufficient information about their conditions and treatment plans [28]. Enhancing communication with patients—particularly when discussing their intentions—can lead to better perceptions of ambulance personnel. Patients may see this enhanced communication as a sign of respect, potentially reducing the incidence of workplace violence.

Previous research has also described other trigger points that may increase the risk of workplace violence directed toward ambulance personnel [16, 17, 29], including the behaviours of ambulance personnel themselves [25, 27]. To potentially reduce the occurrence of aggressive behaviours, it is essential to understand why aggressive behaviour occurs in the first place [25]. For example, police presence in an aggressive situation may provide a sense of security for ambulance personnel, but it has also been shown to create additional challenges and even provoke aggression in some cases [25].

This study contributes to the research by providing more insight and understanding on workplace violence within the healthcare system [19]. From the personal perspective, studies have indicated an increased risk of workplace violence when patients suffer from mental illness or are under the influence of alcohol or drugs [1, 8, 30, 31]. Additionally, men are the most common offenders [8]. Studies on contextual components reveal that workplace violence is more prevalent if patients have to wait for the ambulance to arrive [32, 33]. This is also evident in cases where patients are in public places, such as bars or restaurants, particularly during weekends [8]. From a contextualized structural perspective, research suggests enhanced cooperation between ambulance personnel and police enforcement [34] as well as the importance of training programs on the de-escalation of aggressive situations [31, 35]. The findings of this study contribute to the body of research in various ways. From the personal perspective, ambulance personnel may gain insight into how their behaviour is perceived. However, from the contextual and structural perspectives, the findings highlight the role of factors like wait times, differing expectations, and collaboration with other emergency services (i.e., police enforcement).

The current study reveals how the behaviour of ambulance personnel may be perceived by patients—in this case, the offenders—and how such behaviour may trigger workplace violence. The current study indicates that vulnerable patients who feel disrespected and misunderstood by ambulance personnel may signal a risk for workplace violence. Additionally, if expected and provided care do not match, patients may direct their aggressive behaviour towards ambulance personnel. Further investigation should be done regarding understanding patient and society expectations for ambulance care. In doing so, ambulance personnel may be able to further minimize the mismatch between expectations and reality.

### Limitations

To our knowledge, this study represents the first use of trial transcripts to explore the offender’s perspective in the context of workplace violence directed toward the ambulance service. This qualitative methodology employed provided insight into why the offender acted in the way they did. However, the use of trial transcripts contains the risk of selection bias, since only convicted offenders were included, and data are not primarily collected from the offenders themselves, as in interviews. However, reviewing trial transcripts enabled access to relevant data that was publicly available, relatively easy to obtain, and established by a court decision. Also, this study did not include less serious offenses or trials in which the defendant was not found guilty. Notably, criminology research has successfully utilised trial transcripts to study offender perspectives [36, 37]. Therefore, the current study sample and data collection strategy was considered time- and cost effective.

However, the study design also had its limitations. The results of this study are based on trial transcripts in which the offenders were convicted of a crime and were likely to be defending their actions. With that in mind, it is important to view these results critically.

Some offenders were under the influence of alcohol or drugs during the incident. While this may have affected their recollection of it, it is also possible that these offenders fabricated parts of their testimony as a form of self-defence.

Over 15,000 trial transcripts were reviewed to identify those eligible for inclusion in this study. However, some transcripts may have been overlooked during this extensive review process. In addition, the included transcripts do not represent all events of criminal acts directed toward the Swedish ambulance service during the ten-year period. With that said, it is our belief that information power has been met in this study, and we base this on the following criteria [38]: the narrow objective of the study, the selection of study participants, the structured data collection with its broad inclusion criteria, the structured and internally validated analysis, and the research team’s clinical and scientific expertise regarding the phenomena studied.

Given that only convicted offenders were included, the transferability of this study can be questioned. It can be argued, however, that the purposive sampling method used strengthened the study. This is supported by confirmation that the participants were found guilty by the district courts. This is also supported by the certainty that the plaintiffs were ambulance personnel or the ambulance service as an organization. To further allow for transferability to other contexts, the sampling process, the offenders and crimes committed are described in detail (39).

## Conclusion

From the offender perspective, workplace violence towards ambulance personnel or the ambulance service was the result of: a mismatch between expectations, misunderstandings, feelings of being disrespected, and being in a vulnerable state. These factors were noted as possible triggers for aggression. Educators and training programs can potentially use this insight into offender perspectives of workplace violence to mitigate such violence in the ambulance service context.

## Data Availability

The data that support the findings of this study are available from the corresponding author (MV) upon reasonable request.
